# Recent Advances, Challenges, and Functional Applications of Natural Phenolic Compounds in the Meat Products Industry

**DOI:** 10.3390/antiox14020138

**Published:** 2025-01-24

**Authors:** Ting Bai, Xiulian Wang, Wenqing Du, Jie Cheng, Jiamin Zhang, Yin Zhang, Roungdao Klinjapo, Suvaluk Asavasanti, Patchanee Yasurin

**Affiliations:** 1 Meat Processing Key Laboratory of Sichuan Province, Chengdu University, Chengdu 610106, China; 2 Theophane Venard School of Biotechnology, Assumption University, Bangkok 10240, Thailand; 3 College of Food and Biological Engineering, Chengdu University, Chengdu 610106, China

**Keywords:** natural phenolic compounds, antioxidation mechanism, antibacterial mechanism, pro-oxidant, meat products industry

## Abstract

Natural phenolic compounds (NPCs) have been proven to effectively extend the storage time of meat products in recent years. To promote the discovery of more NPCs and their applications, this review examines recent progress in the classification, antioxidant, and antibacterial mechanisms of NPCs used in meat products. These compounds are found in both edible and inedible parts of plants, including fruits, vegetables, and trees. The recycling of agricultural by-products aligns with green agricultural trends and serves as a guideline for developing new sources of natural additives. Studies on the application of NPCs in various livestock and poultry products, either directly mixed into the matrix or indirectly contacted by preparation into bioactive films and packaging materials, has highlighted the great potential of NPCs. The pro-oxidative effects of NPCs on proteins and their interactions with biological macromolecules, such as proteins, provide new ideas for in-depth research on antioxidant and antibacterial mechanisms.

## 1. Introduction

Pork, beef, chicken, rabbit meat, and other livestock and poultry are the major sources of meat for human consumption. These meats are rich in nutrients, such as protein and fat, but they are prone to oxidation and deterioration during processing, air-drying, and storage, affecting consumer acceptance [1,2,3,4]. Traditionally, synthetic antioxidants and antibacterial agents, such as propyl gallate (E-310), butylated hydroxyanisole (BHA; E-320), and sodium nitrite (E-250), have been used to prevent meat and meat products’ spoilage [5]. However, there are concerns about their possible toxicological effects and cancer risks [6]. Consequently, natural preservatives are increasingly used as alternatives to synthetic ones [7]. For instance, rosemary and oregano are approved for use as spices by the European Food Safety Authority (EFSA), meeting the food industry’s needs to reduce or eliminate the use of synthetic food additives, improve consumer acceptance, and reduce potential health risks [8,9]. NPCs derived from plants or plant by-products have received considerable interest from meat producers, additive companies, and researchers [10].

The global market for plant extracts reached USD 31.14 billion in 2024, and is projected to reach USD 615 billion by 2031 [11], in which fruit waste and agricultural by-products are considered as potential sources of natural additives, supporting a green, circular economy [12,13]. The assessment of NPCs from 20 types of fruit, including tropical, subtropical, and temperate varieties, has shown beneficial antioxidative and antibacterial functions, as well as improvements in color and flavor [14].

NPCs are natural, efficient, and environmentally friendly ingredients commonly used in the food industry, particularly in clean-label foods [15,16]. Leveraging the bioactivity of NPCs can address issues associated with the production and preservation of meat and meat products by replacing synthetic additives [17]. To achieve the best preservation effect, research focuses on exploring new sources of plant extracts, identifying bioactive components, developing methods to maximize bioactivity, and evaluating preservation effects and mechanisms [18,19,20]. With advances in active packaging materials and nanotechnology, increasingly more studies are focusing on composite and nanoencapsulation active films (coatings). Composite films prepared by adding pine needle extracts containing cedar polyphenols showed a strong free radical scavenging ability, shown through the inhibition results of fat and protein for the model of high-fat cured meat [21]. Nanotechnology can significantly improve the activity of naturally existing substances. Green tea extract, horse berry extract, amaranth leaf extract, and various nanoliposome packaging materials containing active plant ingredients have showed strong antioxidant and antibacterial properties [22].

Most recent research on NPCs used in the meat industry has aimed at identifying new sources of NPCs and extracts, revealing synergistic effects and improving the understanding of the underlying mechanisms. This review aims to provide an overview of the main sources, compositions, and functions of NPCs, a list of their applications and effectiveness as antioxidants and antimicrobial compounds in meat and meat products, and a summary of the antioxidant and antibacterial mechanisms. The emphasis is on the pro-oxidative effect of NPCs on proteins, as well as the phenomenon of interactions with proteins or other biomacromolecules affecting antioxidant and antimicrobial effects, providing a reference for further research on the roles and mechanisms of NPCs in meat and meat products.

## 2. Natural Phenolic Compounds and Their Functions

### 2.1. Natural Phenolic Compounds Identified in Plant Extracts

NPCs are secondary plant metabolites with at least one aromatic ring and two hydroxyl groups. They primarily include phenolic acids, flavonoids, glycosides, terpenoids, and their derivatives, depending on their chemical structures [23,24]. NPCs are abundant, diverse, and widespread, arising from both edible and nonedible plant materials, such as vegetables, fruits, medicinal plants, grains, woody resources, bean sprouts, and roots [25,26]. NPCs are present in various plant parts, including leaves, turbs, peels, seeds, roots, husks, and fruits, as well as by-products of processing, such as wastewater and slag (Table 1). NPCs are concentrated forms of natural bioactive compounds that can be effective in small amounts. To leverage their biological activity and high added value, effective extraction technology, the effective use of agro-industrial by-products, and environmentally friendly, sustainable green processing technologies are future priorities [27,28].

### 2.2. Functions of Natural Phenolic Compounds

The chemical structure of NPCs influences the diversity of their biofunctional properties, including antioxidation, antibacterial, anti-inflammatory, and certain pharmacological properties, making them potentially useful in both the pharmaceutical and food industries [24]. In the meat products industry, they are applied to ham, dry fermented sausage, and frankfurter sausages to inhibit microbial growth, delay lipid rancidity, inhibit myoglobin oxidation, and stabilize meat color [49,50]. Tea polyphenol/kojic acid chitosan nanoparticle films prepared using the ion gel method significantly inhibited microbial spoilage of air-dried chicken [51]. In addition to antioxidation and antibacterial effects, 1% *Alpinia katsumadai Hayata* (containing cardamonin, alpinetin, and pinocembrin) and 0.01% curcumin can inhibit acrolein, which is highly toxic to cells during meat heating [52], and slightly higher levels of quercetin also maintain the gel properties of myofibrillar proteins (MPs) by influencing moderate crosslinking and aggregation of MPs resulting from covalent and noncovalent connections [51]. A mulberry polyphenol extract with 312.3 ± 11.67 mg gallic acid equivalent per gram significantly improved the pork MP digestibility and antioxidant activity of digestive fluids [53]. Extracts from broccoli, asparagus, and ginger have also improved beef tenderness [54]. The sensory acceptability of aged mutton can be enhanced with the application of crude water extracts from *Menthapiperitae folium* and *Zingiber officinale*. In particular, this improves the overall sensory properties and fatty acid composition while eliminating the adverse lamb flavor and producing a more stable color and high tenderness score [55]. As a result, NPCs are increasingly prominent as food additives due to the increasing functional properties.

Research on the effect of “natural antioxidants” or “natural antibacterial agents” on meat or meat products is obviously increasing, as the application of NPCs can improve the functionality of meat products and produce clean-label foods that meet consumer demands [27]. However, there are research and application limitations. The source of these natural additives is crucial, considering factors like cost, effectiveness, and potential toxicity. Higher amounts of antioxidants can be harmful due to their tendency to oxidize easily [56]. NPCs are generally less active than synthetic ones and their effectiveness largely depends on the food matrix, as the unsaturation level of fatty acids in different meat matrices and the interaction between meat components (such as lipids and proteins), as well as the difference in the rate of change of fatty acid composition in different matrices during storage, can affect the activity of NPCs [57]. Therefore, there are still many areas that need breakthroughs in the application and mechanism research of NPCs.

#### 2.2.1. Antioxidation Effect

Numerous studies have focused on the application of NPCs on different kinds of meat and meat products (Table 2), including fresh meat, sausage, hamburger, patties, paste, meatball, dry-cured meat, slices, and others from pork, chicken, beef, duck, lamb, and rabbit. NPCs can inhibit lipid–protein oxidation, prevent the development of adverse flavors, improve the sensory properties of products, and more by directly adding phenolic monomers, mixtures, or complex compounds, or indirectly by preparing into bioactive membranes or packaging bags [58,59,60].

In terms of direct addition, monomer compounds like phytic acid and tea polyphenols were employed in chicken wings. The anti-free-radical properties of polyphenols can inhibit the oxidation of carbonyl derivatives that affect meat flavor. They can also significantly impact the flavor of seasoned chicken wings, and significantly reduce some volatile compounds [61]. The addition of chlorogenic acid can significantly inhibit lipid oxidation in roasted lamb [62]. Natural phenolic mixtures or complex compounds are mainly derived from plant extracts. For example, phenolic acids and flavonoids isolated from Olive (*Olea europaea* L.) leaves and olive by-products (wastewater and pomace) can be used as natural food additives to improve safety and quality [63,64,65]. The bioflavonoid complex from black chokeberry and black currant extracts can delay fat peroxidation in smoked sausage storage, and 0.2–0.5% black bilberry extract can stabilize oxidation by-products in high-fat smoked sausages [66]. Proanthocyanidins were isolated from litchi peel using optimized liposome encapsulation technology and they demonstrated much stronger antioxidant activity than the control group (which only contained oligomeric procyanidins and not encapsulation) [67], while films containing a mixture of pectin and polyphenols from watermelon peel considerably reduced TBARs and TVB-N [68]. Baobab seed (*Adansonia digitata*) extract containing NPCs of terpenes and flavonoids exhibited antioxidant properties against beef patties [43]. In addition to mixing directly with minced meat, the meat can also be soaked in a preservative solution. Chilled pork meat was soaked in a preservation solution prepared from sea buckthorn seeds, exhibiting significant antibacterial properties against *Staphylococcus aureus* (*S. aureus*) and the capacity to scavenge hydroxyl free radicals (·OH), 2,2′-Azinobis-3-ethylbenzthiazoline-6-sulphonate (ABTS), and 1,1-diphenyl-2-picryl-hydrazyl radical (DPPH), equivalent to that of VC (*p* < 0.05) [69].

On the other hand, NPCs can be employed as natural additives or active components in films to limit lipid–protein oxidation and microorganism development during production and storage, thus extending the shelf life [17,70]. For example, active films containing watermelon rind polyphenols or rosehip extracts had better mechanical properties and significantly reduced TBARs in chilled lamb and chicken breast, respectively [58,68]. Active films treated with nanotechnology have better physical properties, biological activity, and stability and are receiving more attention. For instance, nanoemulsion films loaded with curcumin polyphenols showed antimicrobial activity against *Salmonella typhimurium* (*S. typhimurium*) and *Escherichia coli* (*E. coli*) and extended the shelf life of fresh chicken to 17 days, which may be attributed to the fact that Gram-negative (G^–^) bacteria are more sensitive to silver nanoparticles [71]. The DPPH free radical scavenging activity of pomegranate peel nanoparticles was significantly enhanced. When applied to pork meatballs, the POV, TBARS, and TVB-N of the product were significantly lower than those of pomegranate peel extract without nanoparticles, as reducing the particle size to nanoscale can increase the surface area and promote the release of phenols [72]. The bioavailability and stability of active edible films loaded with eugenol nanoparticles were significantly improved, extending the shelf life of chicken breast meat without adversely affecting color, texture, and sensory qualities. The free radical scavenging effect of eugenol-containing hydroxyl groups and gelatin-containing aromatic amino acids improved the antioxidant activity, while gelatinase secreted by *S. aureus* accelerated the release of eugenol from the membrane at 37 °C [73]. Therefore, active packaging technology combines barrier properties and bioactivity to improve the bioavailability and controlled release of NPCs. Exploring biodegradable films based on natural biopolymers from plant by-products is the research focus of future food packaging technology [59,74]. Considering that consumers prefer meat-based products with fewer (or no) additives, NPCs are released through active packaging to interact with the food matrix. This indirect addition strategy has greater market prospects in the meat industry.

**Table 2:** The application of natural phenolic compounds as antioxidants in the meat products industry.

Plant Sources	Phenolic Compounds	Object	Processing	Application Effects	References
Sea buckthorn seed polyphenols (SBS)	Prodelidin, epigallocatechin, gallocatechin, and anthocyanidin (epicatechin and catechin)	Fresh pork meat	Soaked in the composite (2.5% free phenol, 2.25% chitosan, and 0.15% VE), single preservation solution (2.5% SBS), and 2.5% tea polyphenols for 15 s. Placed in a sterilized ziplock bag and refrigerated at 4 °C	The maximum scavenging rates of free phenol and bound phenol to DPPH free radicals were 83.14 ± 0.12% and 81.09 ± 0.19%	[69]
Date seed of cultivar Deglet Nour (*Kébili region*) extract	Flavonoids and anthocyanin	Fresh chicken breast	C (no antioxidant), BHT (legal limit of 100 mg/kg), DSEE1 (0.156% (*v*/*w*)), DSEE2 (0.312% (*w*/*v*)), and DSEE4 (0.625 (*w*/*v*)); 14 days at 4 °C	DSEE delayed the primary oxidation; the lower TBARS value was in the DSEE4 sample	[75]
*Lepidium sativum* seed mucilage and *Satureja hortensis* L. essential oil	*Isopropyl Myristate* and *Carvacrol*	Fresh lamb meat	A control sample, mucilage-coated (LSSM), mucilage-coated +0.5% SHEO (LSSM + 0.5% S), mucilage-coated +1% SHEO (LSSM + 1% S), and mucilage-coated + 1.5% SHEO (LSSM + 1.5% S), during storage for 18 days at 4 °C	The lowest TBA and POV levels were for the lamb meat coated with LSSM + 1.5% S	[76]
*Curcuma longa* (CL), *Myristica fragrans* (MF), *Zingiber officinale* (ZO), *Cymbopogon citratus* (CC), and *Thymus vulgaris* (TV), as well as their mixture	Eugenol, turmerone, ar-turmerone, and carvacrol thymol	Fresh rabbit meat	The aqueous extracts (CL, CC, MF, ZO, TV, and mixture) were added (0.2 gm/100 gm rabbit meat), with a negative control (NC, no extracts) and positive control (PC, 0.2 BHT/100 gm sample), and refrigerated at 4 ± 2 °C for 16 days	The lowest TBARS values were detected for the samples treated with 0.2% of the extracts mixture	[77]
Ethanol extract from lotus seed peel powder	Five flavonoids: catechin, epicatechin, rutin, phlorizin, and quercetin; three phenolic acids: gallic acid, ferulic acid, and caffeic acid	Pork sausage	0.10% EEL (LD), 0.15% EEL (MD), 0.20% EEL (HD), and 0% EEL (BC), roasted for 25 min at 80 °C, cooked at 80 °C in a water bath for 20 min, pasteurized with hot water at 85 °C for 2 min, and stored at 25 °C for 18 days	EEL and NaNO_2_ significantly reduced the TVB-N, while the TBARS value of each EEL group did not change significantly in the range of 7.12–8.55 g/100 g	[78]
Grape skin flour (*Vitis vinifera Var. Tempranillo*)	Anthocyanins and tannins	Beef burger	HT (BF + 0.01 g BHT/100 g fat), WBM0.5 (0.5 g WBM/100 g BF), WBM1.0 (1.0 g WBM/100 g BF), WBM1.5 (1.5 g WBM/100 g BF), and WBM2.0 (2.0 g WBM/100 g BF), stored under freezing (−20 ± 2 °C) for 120 days	Used at up to 1 g/100 g to replace BHT as a natural antioxidant in frozen beef burgers	[79]
D.O.*Valle del Jerte* cherries (*Pico negro* variety) extract	-	Lamb burger	C: no natural additive, CH2: 2% cherries (*w*/*w*), CH6: 6% cherries (*w*/*w*), and CH10: 10% cherries (*w*/*w*)	Total antioxidant activity increased with increasing cherry content	[80]
Aqueous coriander extract	-	Chicken patties	BHT (100 ppm) and aqueous coriander extract (1%), refrigerated for 9 days	The formation of peroxides, TBARS, total carbonyls, and metmyoglobin was reduced	[81]
Olive leaf extract (OLE), thyme leaf extract (TLE), and their combination	-	Lamb patties	Untreated (control), 1% olive leaf extract (T1), 0.05% thyme leaves extract (T2), 1% OLE plus 0.05% TLE (T3), and 0.5% OLE plus 0.025% TLE (T4), stored for 12 days at 4 °C or at −18 °C for 120 days	OLE, TLE, and their combination are effective in retardation of oxidative rancidity	[82]
Açaí extract powder	Flavonoids, such as orientin, homoorientin, vitexin, luteolin, chrysoeriol, quercetin, and dihydrokaempferol	Pork patties	No antioxidant (CON), sodium erythorbate 500 mg/kg(ERY), açaí extract: 250 (AEL), 500 (AEM), and 750 mg/kg (AEH), packed in nylon-polyethylene bags, sealed without vacuum, and stored at 2 ± 1 °C for 10 days in the dark	250 mg/kg of açaí extract can be used as a natural antioxidant to decrease lipid oxidation	[83]
Piper chaba (*Khulna*, *Bangladesh*) stem extracts	Catechin hydrate, vanillin, syringic acid, caffeic acid, chlorogenic acid, rutin hydrate, tannic acid, and quercetin hydrate	Beef patties	Control (contained only spices), PEE (contained spices and 0.2% ethanol extract but no BHT), and PCP (contained spices and 0.1% BHT but no ethanol extract), refrigerated at 4 °C ± 1 °C for 33 days.	The fat content of the PEE and PCP degraded at a slower rate than the control, and both PCP and PEE had increased antioxidant capacity	[84]
Manuka (MO), rosemary (RO), and kānuka (KO) oils	-	Beef paste	MO 1, 2, and 3 (5%, 25%, and 40%), RO, and KO	The MO addition led to a significant reduction of lipid oxidation	[85]
Olive (*Olea europaea* L.) leaf extract	-	Mutton meatball	T0 (0), T1 (0.1), T2 (0.2), and T3 (0.3%), respectively, based on olive leaf extract supplementation, preserved at 4 °C for up to 10 days	0.3% olive leaf extract is suitable to act as a source of natural antioxidant	[86]
Clove essential oil (CEO) using nanoemulsions	-	Chicken meatball	No CEO (C), 10 mL nanoemulsion containing 2.5% CEO (T1), and 15 mL nanoemulsion containing 2.5% CEO (T2), boiled in steam at 80 °C, packaged in LDPE pouches, and then stored at 4 °C	The increase in TBARS values in the T1 and T2 groups was significantly lower than that in the control group	[87]
Tea polyphenol (TP), apple polyphenol (AP), and cinnamon polyphenol (CP)	-	Dry-cured bacon	Additives (AA, TP, AP, and CP) with different concentrations (0, 100, 300, and 500 mg/kg), pre-heated at 200 °C, and roasted for 90 s without oil, then frozen at −25 °C until analysis	Bacon containing 300 mg/kg AP produced less TBARS and carbonyl contents	[88]
Clove extract	Eugenol, fatty acids, and flavonoids	Dry-cured duck	Traditional dry-cured duck (3% salt), with the clove treatment (3% salt and 0.1% clove extract), stored for 180 days	The POV and acid value declined significantly, and the shelf life extended to 6 months	[89]
Mulberry polyphenols (MP)	Anthocyanins and flavonoids	Pork slices	1.01 g MP per kg mixture (68 mg C3GE/kg), without MP as a control, vacuum-packed and stored at room temperature for 7 days	MP delays the formation of carbonyl and S-S groups	[90]
Black garlic extract (Haenafood Co. (Seoul, Republic of Korea))	-	Cooked chicken breast	12 treatment groups containing fresh BG extract (1:4, *w*/*v*; positive control), distilled water (negative control), oven-dried BG, and encapsulated BG extract	Maltodextrin-encapsulated extract prolonged the protection of the antioxidant BG compounds	[91]

#### 2.2.2. Antibacterial Effects

NPCs are antibacterial agents shown to inhibit food spoilage by microorganisms and food-borne pathogens [23]. Previous studies also focused on the antimicrobial and antifungal effects of NPCs on different kinds of meat and meat products (Table 3). Basil, thyme, and tarragon had antibacterial effects against *Salmonella Abony* in sausage, where the highest inhibition was caused by basil (97%), followed by tarragon (95%), with thyme showing the smallest decrease (90.2%) [92]. *Ephedra alata* aqueous extract (EAE) is rich in flavonoids and anthocyanins, and has shown antibacterial activity against *E. coli* ATCC 8739, *S. aureus* ATCC 6538, *Listeria monocytogenes* (*L. monocytogenes*) ATCC 19117, and *Salmonella enteric* ATCC 14028, with minimum inhibitory concentrations (MICs) of 3.12, 1.56, 1.56, and 3.12, respectively. Mangosteen peel extract was most effective in reducing *E. coli* and *S. aureus* [19]. However, when applied to minced beef refrigerated for two weeks, it showed different antibacterial efficiency in situ, where the effect of 0.624% EAE (equivalent to 4 × MIC) on the Enterobacteriaceae count was not significantly different from that of 0.156 and 0.312 EAE (*p* > 0.05). This may be due to the interaction of proteins or fatty acids with ingredients in *Ephedra alata*, affecting the cell membrane structure and function [93,94].

## 3. Mechanisms

### 3.1. Antioxidative Mechanism

#### 3.1.1. Lipid

**Figure 1:**
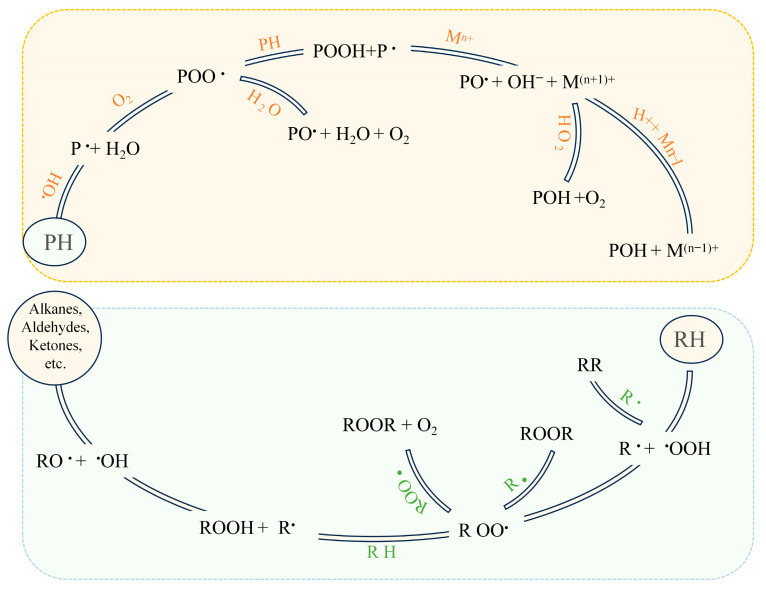
The mechanism of general protein (yellow background) and lipid (green background) oxidation [2].

**Figure 2:**
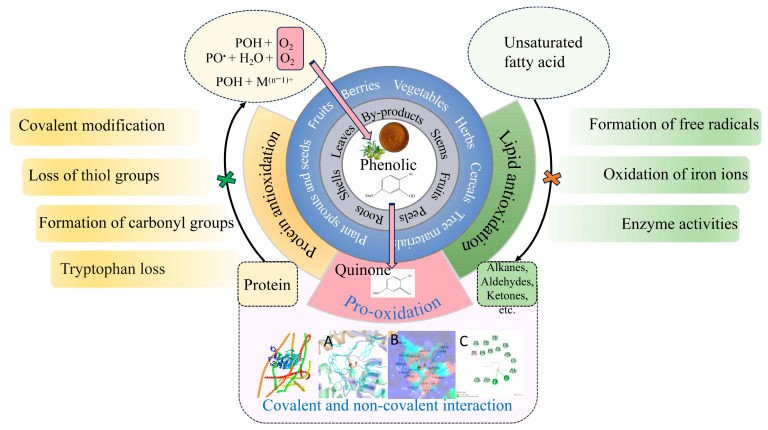
The sources of natural phenolic compounds and their antioxidant and pro-oxidant effects on pro-teins and lipids. A: Molecular docking complex conformation; B: 3D interaction map; C: 2D interac-tion map; 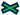: Inhibitory effects of NPCs on protein oxidation; 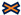: Inhibitory effects of NPCs on lipid oxidation.

The free radical mechanism (Figure 1) is the primary process of lipid oxidation that occurs in meat and meat products, typically involving the interaction between molecule-level oxygen and unsaturated fatty acids, and the metabolites involved in the reaction include fatty acids as precursor compounds, hydroperoxides as the primary metabolites, and small molecular volatile flavor compounds as the secondary metabolites [1]. Lipolysis increases lipid oxidation, decreasing product quality and promoting flavor formation to some extent, leading to a conflicting effect of lipolysis on lipid oxidation in meat and meat products [96]. The release of free radicals and the action of metal ions and enzymes are the main factors affecting lipid oxidation. NPCs can be used as natural antioxidants to remove free radicals and reactive oxygen species, decrease the generation of oxidation metabolites, and act as metal chelating agents (such as iron and copper ions). In terms of enzyme inhibition, they can inhibit various enzymes, including free-radical-forming oxidases, peroxidases, antioxidant enzymes activated by peroxidases, and superoxide dismutase [23] (Figure 2). Direct multi-hydrogen and hydrophobic bonding can achieve the inhibitory effect of polyphenols on proteases to affect the structure of protease and the activity of lipid-oxidation-related enzymes [97].

The sensitivity of meat to oxidation is more related to unsaturated fatty acids than to total fat content; for example, polyunsaturated fatty acids in meats, such as chicken and beef, can be oxidized to different hydroperoxides, which are further degraded into different small-molecule compounds [98,99]. The presence of NPCs can significantly influence the variation in fatty acid composition of inner muscle fat throughout meat preservation, but it mostly depends on the meat matrix [57]. In addition, NPC modification of the protein structure, especially changes in the secondary structure, affects the adsorption and release of lipid oxidation products (Figure 2). For instance, adding mushroom polyphenols to beef can enhance the interaction between sarcoplasmic proteins with lipid oxidation products, thereby reducing the flavor compounds after lipid oxidation [100]. Green tea phenolic compounds can bind near His 64 on the myoglobin surface, blocking the potential combination of aldehydes with this specific histidine [101].

The antioxidant effects—single or synergistic—depend on the nature of the active molecules. Different extracts with similar mechanisms of action, like grape seed and pine bark extracts containing NPCs, such as polyphenolic acid, caffeic acid, catechins, epicatechins, and resveratrol, can significantly improve antioxidation of cooked beef by scavenging free radicals [102]. One extract may have multiple mechanisms of action. Oleuropein and oleuropein derivatives with hydroxytyrosol as the main component in olive processing wastewater by-products, quercetin, luteolin, 7-glucoside, and phenolic aldehydes in olive leaves, as well as hydroxytyrosol in olive dregs, can decrease the presence of transition metals and free radical scavenging effects [103,104,105]. Therefore, direct addition of an olive leaf extract can significantly inhibit the oxidation and microbial activity in patties and meatballs [82,86].

#### 3.1.2. Proteins

The pathway of protein oxidation is similar to that of lipid oxidation, a free radical chain reaction (Figure 1), but more complex and with more oxidation products. It is a covalent modification caused by the change in the protein structure after lipid peroxidation induced by oxidative stress active compounds (H_2_O_2_, metal catalysts such as Fe and Cu, and reactive oxygen species). This change involves the irreversible synthesis of carbonyl groups and the reversible removal of thiol groups [106]. Thiol groups are targets of protein oxidation in meat, producing sulfur-containing compounds, such as disulfides and sulfenic acid; further, the interaction between protein side chains and secondary metabolites, such as ketones and aldehydes, also modifies MPs, affecting their functional properties [107]. Lysine oxidation products, particularly α-aminoadipate and Schiff bases, as well as the loss of tryptophan, can be used to assess protein oxidation [108,109].

The antioxidative mechanism of NPCs on proteins is similar to that on lipids, but not exactly the same, since NPCs have a pro-oxidative effect on proteins (Figure 2). NPCs have pro-oxidative activity, which indicates the complexity of the antioxidative mechanisms for proteins. Bologna-type sausages with green tea or rosemary extract had a lower content of TBARS and protein carbonyls. In contrast, the increase in thiol loss was inhibited by a green tea extract. NPCs can be oxidized to form quinone compounds, which interact with protein thiol groups, enhancing protein polymerization to produce phenol-mediated protein polymerization, and free radical strength increases in sausages, attributed to protein-bound phenoxy free radicals that prevent other oxidation-induced protein modifications [56,110]. Gallic acid is considered to be an oxidant of tryptophan, and quercetin promotes the oxidation of tryptophan, which promotes the oxidation of pork patties [111,112]. In addition, rosemary had a significant effect on scavenging free radicals in a pork model system, as well as pro-oxidant effects on thiols in the OXHydro system [107]. In general, strong oxidation-promoting effects are only found with high-dose phenolic compounds [110].

On the other hand, interactions between NPCs and proteins may modify the protein structure and function. For instance, in the electrostatic interaction between quercetin and quercitrin with myofibrillar protein (MP), the hydrogen bonding between tannic acid and gallic acid with MP enhanced the oxidative stability of proteins and significantly improved the texture and antioxidant properties of pork meatballs [113]. Molecular docking results also showed that hydrogen bonding, hydrophobic interaction, and electrostatic interaction were the main molecular forces between phenolic compounds in pine needle extract (PNE) from *Cedrus deodara* and salted bacon myofibrillar protein [114]. Similarly, the interaction between chlorogenic acid and lipoxygenase is mainly manifested by hydrogen bonds, hydrophobic interactions, and van der Waals forces [115]. The hydroxyl groups on NPCs can serve as hydrogen donors to interact with amino acid residues in proteins, and the number of hydroxyl groups significantly affects the biological activity of NPC–protein complexes [116,117].

### 3.2. Antibacterial Mechanism

The antibacterial mechanism of NPCs mainly involves destroying cell membranes and cell walls, inhibiting DNA synthesis, affecting enzyme activity, interfering with energy metabolism, and the effect of free radicals on reactive oxygen species (ROS). For example, phenolic compounds from black currant (*Ribes nigrum* L.) achieved bacteriostasis against *S. aureus*, *E. coli*, and *S. typhimurium* by inhibiting biofilm formation and DNA synthesis, destroying cell walls and membranes [118]. Rosmarinic acid (RosA) exhibited antibacterial activity against *E. coli*, *S. aureus*, *Salmonella*, and *Bacillus subtilis* by destroying bacterial cells and cell proteins and inhibiting the activity of intracellular Na/K-ATP-ase [119]. The antibacterial mechanism of hawthorn extract against *S. aureus* includes inhibiting intracellular enzyme activity, destroying the integrity of the cell wall and cell membrane, increasing ROS, and changing the expression of related genes [120]. Other studies have found that the interaction between flavonoids and isoflavonoids in red propolis extract and amino acid residues in the casein matrix enhanced the absorption of active compounds by biofilm and exhibited antibacterial activity against *S. aureus* and *Pseudomonas aeruginosa* [121].

However, among the antibacterial mechanisms based on the interaction between NPCs and biomacromolecules, such as cell membrane proteins, lipid bilayers, and ATP synthase, hydroxyl and hydrophobic interactions are the most focused on. The interactions between polyphenols and cell membranes include hydrogen bonds and hydrophobic interactions, in which the hydroxyl groups have the possibility to act as hydrogen bond donors or acceptors [122]. Green tea catechins formed hydrogen bonds and hydrophobic interactions with the target bacteria, showing antibacterial activity [20]. Dietary pomegranate phenolics bound to the ATP synthase in *E. coli* and significantly reduced the enzymatic activity, affecting microbial metabolism [123]. Dietary ginger powder (DGP) interacted with residues of the α, β, and γ subunits of ATP synthase to inhibit its antimicrobial activity [124]. The increased hydrophobicity of modified carboxylic acid can enhance its interaction with the hydrophobic lipid cell membrane on *E. coli*, improving its antibacterial activity [125]. The antimicrobial activity of NPCs against G^+^ and G^−^ bacteria is related to hydroxyl groups in the lipid bilayer of the lipophilic outer membrane, which was confirmed in the study of six phenolic acids against *Lactobacillus rhamnosus* and *E. coli* [126]. In addition, the interaction between carvacrol and myoglobin led to a decrease in the antibacterial activity of carvacrol, and hydrogen bonding was the main force in the interaction [127].

The number of hydroxyl groups and the polymerization degree in polyphenol structures appear to have a direct correlation with antimicrobial activity. For instance, the difference in the number of hydroxyl groups in White Wormwood (Artemisia herba-alba) extracts resulted in different antimicrobial activities against G^+^ (*Bacillus cereus* and *S. aureus*) and G^–^ (*E. coli* and *Proteus vulgaris*) microorganisms [128]. Four bioflavonoids from fruits of the Brazilian peppertree (*Schinus terebinthifolius* Raddi) have diverse antimicrobial activity due to the diverse connection modes of flavonoids and the saturation degree of the C-ring. In particular, this shows the importance of methoxyl or hydroxyl groups in microbial inhibition [129]. The inhibitory effect of date palm seed extracts on target bacteria may be related to the quantity and orientation of hydroxyl compounds in the phenol ring, causing membrane disruption and/or metal chelation by flavonoids [75].

## 4. Conclusions

NPCs or extracts from various sources, including roots, stems, leaves, and other parts of fruits, vegetables, and trees, offer significant antibacterial, antioxidant, and other bioactive properties. Using fruit peels and other agricultural waste as sources provides market solutions to reduce application costs and aligns with green agricultural practices, as biodegradable and bioactive packaging based on plant and processing by-product sources is more in line with market demand. As natural antioxidants, NPCs inhibit protein and lipid oxidation by scavenging free radicals, removing metal ions, and slowing down enzyme activity, and high doses of active molecules can promote protein oxidation. They also act as natural antibacterial agents by disrupting cell membranes, chelating metal ions, inducing cell death, and chemically reacting with bacterial proteins or enzymes. In addition, the observed interactions between NPCs with myofibrillar proteins affect the functional properties of proteins, while the interaction with biomacromolecules (membrane proteins, lipid bilayers, ATP synthase, etc.) affects the antimicrobial activity. In particular, the presence of hydroxyl groups and their role in hydrophobic interactions or hydrogen bonds have not been clearly elucidated. Therefore, understanding the potential mechanisms of interactions between NPCs and biomacromolecules, such as proteins, remains a considerable area of research.

## Figures and Tables

**Table 1:** Natural phenolic compounds identified in plant extracts.

Sources		Phenolic Compounds	Reference
Name	Part		
Oregano (*Origanum vulgare* ssp. Hirtum)	Flowering aerial part	Phenolic acids (rosmarinic, chlorogenic, cinnamic, caffeic, syringic, benzoic, vanillic, gallic, chicoric, and 2,4-dihydroxybenzoic acids), flavonoids (quercetin, apigenin, luteolin, naringenin, and kaempferol), and coumarin	[18]
Cistus creticus	Flower	Quercetin and gallic acid	[29]
*Flacourtia flavescens* Willd	Leaves	Caffeic acid, apigenin, luteolin, kaempferol, quercetin, gyrophoric acid, luteolin-7-*O*-β-D-glucopyranoside, luteolin-4′-*O*-β-D-glucopyranoside, kaempferol-7-*O*-α-L-rhamnopyranoside, kaempferol-3-*O*-β-D-glucopyranosyl-(1→6)-*O*-α-L-rhamnopyranoside, and kaempferol-3,7-*O*-α-L-dirhamnopyranoside	[30]
*Inga* stipularis *DC.* (*fabaceae*)	Leaves	Ucryphin, neoastilbin, astilbin, neoisoastilbin, isoastilbin, quercitrin, engeletin, and isoengeletin	[31]
*Fagus sylvatica* (European beech)	Leaves	Hydroxycinnamic acids (ferulic, caffeic, and *p*-coumaric acid esters) and flavonoids (apigenin, kaempferol, naringenin, quercetin derivatives, 3″,6″-di-O-coumaroylkaempferol derivatives, chrysin, taxifolin, and (epi)catechin derivatives)	[32]
Sweet potato (*Ipomoea batata* L.; *Lam.*)	Leaves	Phenolic acids: caffeoylquinic acids, esculin, protocatechualdehyde, CA, 7-hydroxycoumarin, and ethyl caffeate	[33]
		Flavonoids: isomeric caffeoylquinic acids, esculin, protocatechualdehyde, CA, 7-hydroxycoumarin, and ethyl caffeate’	
	Tubers	Phenolic acids: caffeic acid, chlorogenic acid, and caffeoylquinic acid derivatives	[34]
		Flavonoids: quercetin, myricetin, luteolin, kaempferol, apigenin, and anthocyanins	
*Dioscorea persimilis*	Tubers	2,4,6,7-tetrahydroxy-9,10-dihydrophenanthrene, aerosin, gastrodin, 2-phenylethyl-β-d-glucopyranoside, afzelechin, catechin, eucomic acid, and vanillic acid (VA)-4-*O*-β-d-glucopyranoside	[35]
Olive	Processing wastewater	Phenolic acids: oleocanthalic acid, caffeic acid, *p*-coumaric acid, ferulic acid, and VA Flavonoids: hydroxytyrosol, tyrosol, oleocanthal, vanillin, verbascoside, luteolin 7 glucoside, pinoresinol, oleuropein, oleacein, and 1-acetoxypinoresinol	[36]
Pomegranate	Peels	Flavonoids (anthocyanins, catechins, and other complexed flavonoids), hydrolyzable tannins (punicalin, pedunculagin, punicalagin), gallic and ellagic acids	[37]
Ginger	Peels	Zingerone, rutin, quercetin, naringenin, kaempferol, and 6-gingerone	[38]
Tomato (*Solanum pimpinellifolium* PI365967 and *S. lycopersicum* Moneymaker)	Fruits	4 chlorogenic acid isomers, caffeic acid-3-*O*-glucoside, dihydrosinapic acid-4-*O*-glucoside, quercetin-5-sophoroside, quercetin-3-*O*-arabinoside-5-*O*-rutinoside, isorhamnetin-3-*O*-glucoside-5-*O*-salicylate, caffeic acid 3-sophoroside, naringenin-5-*O*-glucoside, naringenin-7-*O*-galactoside, and naringenin-4′-*O*-glucoside	[39]
Mulberry (*Morus alba*)	Fruits	Cyanidin-3-*O*-glucoside, cyanidin-3-*O*-rutinoside, rutin, isoquercitrin, resveratrol, and caffeic acid	[40]
Chia (*Salvia hispanica* L.)	Seeds	Apigenin 4′-*O*-glucoside and rosmarinic and caffeic acid	[41]
Avocado	Seeds	Flavonoids (luteolin and quercetin), phenolic aldehyde (ethylvainillin and vanillin), and phenolic acids (phthalic acid, ferulic acid, salycilic acid, and *p*-coumaric acid)	[42]
Baobab (*Adansonia digitata*)	Seeds	Terpenoids, sterols, flavonols, and vitamins	[43]
Tea (*Camellia sinensis* L.) *O. Ktze.*	Seed oil	Naringenin, 3,4-dihydroxyphenyl glycol, gentisic acid, hydroxytyrosol, 7-hydroxy coumarine, homovanillic acid, pyrocatechol, p-coumaraldehyde, p-hydroxy phenylacetic acid, and *trans*-cinnamic acid	[44]
Hemp (*Cannabis sativa* L.)	Seeds	4-hydroxybenzoic acid (4-HBA), VA, protocatechuic acid (PA), syringic acid (SGA), and ellagic acid (EA)	[45]
*Rumex* *dentatus* L.	Roots	Musizin-8-*O*-*β*-D-(6′-*O*-malonyl-3″-methoxy) glucopyranoside, 2-acetyl-3-methyl-1,4-naphtho-quinone-8-*O*-*β*-D-glucopyranoside, (2′*R*)-7-hydroxy-2-(2′-hydroxypropyl)-5-methyl acetate chromone, and 2,8-dimethyl-3,6-dihydroxyxanthone	[46]
Coffea Arabica variety Caturra and Catuaí	Husks	Flavonoids, such as anthocyanins (cyanidin-3-glucoside and cyanidin 3-o-ruthinoside), and phenolic acids, such as chlorogenic acid	[47]
*Rosemary* (*Rosmarinus officinalis* L.)	Shrubs	Phenolic acids (salvianic acid, caffeic acid, rosmarinic acid, and salvianolic acid A), flavonoids (luteolin-7-O-rutinoxide, luteolin-7-glucoronide, hesperidin, luteolin, apigenin, hispidulin, cirsimaritin, genkwanin, and salvigenin), and diterpenes (rosmadial, 7-CH3-rosmanol, carnosol, carnosic, and 12-CH3-carnosic acid)	[48]

**Table 3:** The application of natural phenolic compounds as antibacterial agents in the meat products industry.

Plant Sources	Phenolic Compounds	Object	Processing	Application Effects	References
Sea buckthorn seed polyphenols	Prodelidin, epigallocatechin, gallocatechin, and anthocyanidin (epicatechin and catechin)	Fresh pork meat	Soaked in the optimal composite (2.5% free phenol, 2.25% chitosan, and 0.15% VE), single preservation solution (2.5% sea buckthorn seed free phenol), and 2.5% tea polyphenols for 15 s, placed in a sterilized ziplock bag, and refrigerated at 4 °C	The antibacterial effect of free phenol on *S. aureus* (30.54 ± 0.53 mm) was better than that of bound phenol (22.18 ± 0.04 mm), and significantly different from tea polyphenols (34.45 ± 0.16 mm)	[69]
*Ephedra alata* aqueous extract (EAE)	Phenolic, flavonoid, and anthocyanins	Fresh beef meat	Lot 1 and lot 2 (0.01% BHT) as controls, 0.156% (EAE1), 0.312% (EAE2), and 0.624% (EAE3), stored at 4 °C for 14 days	The addition of EAE led to a significant (*p* < 0.05) decrease in aerobic plate count (APC), psychrotrophic total count (PTC), and *Enterobacteriaceae* count growth rates	[94]
Date seed of cultivar Deglet Nour (Kébili region) extract (DSEE)	Phenolic, flavonoid, and anthocyanins	Fresh chicken breast	C (no antioxidant), BHT (legal limit of 100 mg/kg),DSEE1 (0.156% (*v*/*w*)), DSEE2 (0.312% (*w*/*v*)), andDSEE4 (0.625 (*w*/*v*)); 14 days at 4 °C	PTC decreased significantly with the increment of the DSEE concentration, and the Enterobacteriaceae count (EC) of the treated samples was the lowest	[75]
*Lepidium sativum* seed mucilage (LSSM) and *Satureja hortensis* L. essential oil (SHEO)	*Isopropyl Myristate* and *Carvacrol*	Fresh lamb meat	A control sample, mucilage-coated (LSSM), mucilage-coated +0.5% SHEO (LSSM + 0.5% S), mucilage-coated +1% SHEO (LSSM + 1%S ), and mucilage-coated + 1.5% SHEO (LSSM + 1.5% S) during storage for 18 days at (4 °C)	The coating with 1.5% of *S. hortensis* (LSSM + 1.5% S) showed a noteworthy reduction in total viable counts and was the most efficient for the inhibition of the growth of psychrophilic bacteria	[76]
Cistus creticus extract (Mediterranean region of Turkey, CCE)	Quercetin, gallic acid, rutin trihydrate, and caffeic acid	Beef sausage	Control: 0.02% ascorbic acid and 0.05% sodium ascorbate (C), 0.05% CCE (CC1), 0.07% CCE (CC2), and 0.1% CCE (CC3), cooked at 80 °C and packed in a modified package for 11 weeks at 4 °C	Sausages formulated with 0.1% Cistus extract had the lowest microbial growth during the storage period and improved the shelf life by 1 week	[29]
Cinnamon powder (CIN-P)	-	Beef burger	Four treatments (0 g CIN–P, 0.5 g CIN–P, 1 g CIN–P, and 2 g CIN–P/100 g burger) with four storage intervals (0, 7, 14, and 21 days)	At 100 μg/disc, the CIN–P effectively prevented the growth of both G^+^ and G^–^ bacteria	[95]
Ginger (*Zingiber officinale*) peel extracts	6-shogaol, 6-gingerol, quercetin, zingerone, kaempferol, rutin, ferric acid, hyperoside, chlorogenic acid, caffeic acid, and naringenin	Beef patties	Control (no antioxidants), T1: 1% GPE, T2: 2% GPE, and T3: 3% GPE	The GPE-treated samples had a lower total plate count throughout the 24 days of storage	[38]
Aqueous coriander extract	-	Chicken patties	BHT (100 ppm) and aqueous coriander extract (1%), refrigerated for 9 days	Inhibited the growth of microorganisms, and had the lowest total plate count (1.3–2.4 CFU/g)	[81]
Clove essential oil (CEO) using nanoemulsions	-	Chicken meatball	No CEO (C), 10 mL nanoemulsion containing 2.5% CEO (T1), and 15 mL nanoemulsion containing 2.5% CEO (T2), boiled in steam at 80 °C, packaged in LDPE pouches, and then stored at 4 °C	The TVC values of the T1 and T2 samples were lower than the control group	[87]
Clove extract	Eugenol, fatty acids, and flavonoids	Dry-cured duck	Traditional dry-cured duck (3% salt), with the clove treatment (3% salt and 0.1% clove extract), stored for 180 days	The total bacterial colony count declined significantly, and the shelf life extended from 3 months to 6 months	[89]
